# Medical News and Miscellany

**Published:** 1891-04

**Authors:** 


### Medical News and Miscellany.

**Travis County Medical Society Delegates**—Dr. T. D. Wooten, W. A. Morris, J. M. Litten, Q. C. Smith, A. N. Denton.

**At the Post Graduate.**—Drs. W. T. Strain, of Will's Point, and J. M. Fry, of Elmo, are attending the St. Louis Post Graduate Medical College. Dr. Strain is the JOURNAL'S authorized representative.

**Samples Free.**—Messrs. W. R. Warner & Co.'s agent being unable to attend the Waco meeting, has sent a lot of samples o their splendid pharmaceutical preparations to the General Secretary for free distribution. They will be exhibited and labeled: "Take a Sample—Warner & Co.'s Compliments."

---

**Last Warning.**—Let applicants for membership at the Waco meeting of the Texas State Medical Association, April 28th, inst., not forget to *bring diploma*; and let members remember that payment of dues is imperatively necessary. "No dues, no badge," says our sagacious Treasurer, and adds: "Must obey orders if it break owners."

---

**Dr. E. J. Beal**, of Fort Worth, Texas, has received, direct from Dr. Libbitz, of Berlin—the only authorized person to distribute it—a supply of Koch's lymph, and writes the JOURNAL that he will use it shortly in a case of incipient phthisis. Dr. Beal invites correspondence with physicians who have patients who desire to receive the new treatment.

---

**New Made Texas Doctors.**—The closing exercises of the Texas Medical College and Hospital, at Galveston, were held on the 15th ult., Professor West delivering the valedictory on behalf of the faculty. Drs. J. A. Winfrey and W. B. Grace, having completed the required three years courses, and stood excellent examinations were graduated and given diplomas. Dr. Winfrey will locate at Fowler, and Dr. Grace at Iredell, Texas.

---

**Married** in Vicksburg, Miss., by Rev. Henry Sansom, Rector of Christ's church, March 31st, ult., Mr. Robert Patrick Daniel, of Chihuahua, Mexico, son of Dr. F. E. Daniel, of Austin, Texas, to Miss Roberta Banks, daughter of the late Dr. R. Bruce Banks, of Mississippi. Dr. Banks was Jeff. Davis' Regimental Surgeon in the Mexican war, and was also a distinguished Confederate surgeon. Mr. and Mrs. Daniel will be "at home" in Chihuahua, after June 15th.

---

**Small-pox.**—The reports of small-pox in Texas are greatly exaggerated. There is some small-pox at several points, but as a case makes its appearance it is promptly isolated and guarded, and in no instance has it spread beyond the camp. The disease cannot be said to be prevailing. We have received several letters of inquiry as to safety in traveling in Texas. If a person be protected by vaccination there is no danger in going anywhere in Texas. Without vaccination it is not safe to travel at all—anywhere.

---

**Austin District Medical Society** delegates to Waco meeting, 17—(89 members):

Drs. J. A. Davis and J. Cummings, Austin; A. Garwood, New Braunfels; R. P. Talley, Temple; R. Atkinson, San Marcos; W. H. Holloway, Round Rock; F. R. Martin, Kyle; G. W. Christian, Burnet; J. W. Hamilton and J. W. Carhart, Lampasas; L. H. Hardy, Paige; H. H. Thorpe, Liberty Hill; B. E. Hadra, Galveston; W. H. Barnwell, Webberville; S. Cunningham, Elgin; W. T. Richmond, Manor; J. S. Poyner, Bartlett.

---

"**Resolved**—That the general secretary be authorized and empowered to employ a competent stenographer and type-writer, to assist in reporting the discussions and proceedings of the Association at each meeting." Resolution by Dr. West, adopted at Fort Worth, April, 1890.

In pursuance of the above the Secretary has engaged the services of Mr. F. C. Pierce, of Dallas, to attend the meeting at Waco. The Secretary is amply able to take down the proceedings, and has done so, satisfactorily, we believe, for several years; but, not being a short-hand writer, it is impossible for him to secure *verbatim* reports of the discussions—and, as Dr. West remarked, when introducing the resolution, the discussions of papers read would constitute, perhaps, the most interesting part of the proceedings. This being an innovation, this year's transactions may be looked for with more than ordinary interest; the book will be much larger than usual, and containing this, a new feature, will be much more valuable than heretofore. As each member, who "discusses," will be taken down, let the members be careful what they say: it will be like the phonograph, their words will come back to them, just as they were uttered. By the bye, let not the guardians of the treasury be surprised, when it comes to paying for short-hand reports,—they come high,—$15 to $25 a day and expenses.

Successful Cæsarian Operation.—Drs. J. J. and A. D. Burroughs, of Houston, have sent the JOURNAL a photo of a young negro girl, upon whom they performed the Cæsarian section on account of congenital deformity of the pelvis. The operation was successful, and very creditable to the surgeons. The patient had never put her feet to the floor, the legs being flexed and crossed; the arms were raised above her head, and could not be lowered except in sitting position, when they came down naturally to the side. The picture represents mother and babe,—the latter apparently white, lying by the side of the mother, who is a fine type, as to features, of the African negress; the arms being stretched above her head, and the legs crossed; the seat of the incision showing in the focus.

Brief History of the Case.—Lucy H., negress, æt 20, operation March 11, at county hospital, Houston, Texas. Discharged March 24; photo ten days after operation; cause, deformed pelvis. Drs. Burroughs will report the case in detail at the meeting of the State Association at Waco, on the 28th, and doubtless it will appear with illustrations, in the Transactions.

WACO, TEXAS, April 8, 1891.

DEAR DOCTOR:—The following railroads will sell excursion tickets at all principal stations to physicians and families wishing to attend the Texas State Medical Association, which meets in Waco, April 28th, viz: Texas & Pacific; Missouri, Kansas & Texas; Houston & Texas Central; Gulf, Colorado & Santa Fe; International & Great Northern. The rate will be four cents per mile for round trip. The Cotton Belt railroad has, so far, failed to make a rate, but it is hoped that it will in the near future.

The hotels will accommodate guests at the following rates: Hotel Royal, $2.50 per day; Pacific, $2.50 per day; McClelland, $2.50 per day; Cotton Belt, $2.00. Other hotels and boarding houses will accommodate guests at cheaper rates; plenty of room for all. Hoping you will find it convenient to be present and assist in making the meeting a success, I am yours very truly,

H. L. TAYLOR, M. D.,

Committee on Transportation.

